# Towards a Future of Personalized Vaccinology: Study on Individual Variables Influencing the Antibody Response to the COVID-19 Vaccine

**DOI:** 10.3390/vaccines11020217

**Published:** 2023-01-18

**Authors:** Giuseppa Visalli, Antonio Laganà, Daniela Lo Giudice, Sebastiano Calimeri, Daniela Caccamo, Alessandra Trainito, Angela Di Pietro, Alessio Facciolà

**Affiliations:** 1 Department of Biomedical and Dental Sciences and Morphofunctional Imaging, University of Messina, 98125 Messina, Italy; 2 Istituto Clinico Polispecialistico C.O.T. Cure Ortopediche Traumatologiche S.p.A., 98124 Messina, Italy

**Keywords:** COVID-19 vaccination, individual variables, personalized vaccinology

## Abstract

The COVID-19 pandemic has hugely impacted many different aspects of human health, and vaccination is one of the most effective weapons to manage it. However, many different factors, such as age, gender, comorbidities and lifestyles, play a role in the response to infections and vaccines. We carried out this study to evaluate the potential role played by some individual factors in the production of anti-COVID-19 antibodies in the light of personalized and future vaccinology. We conducted an observational study consisting of a retrospective phase, exploiting previous data about anti-COVID-19 antibody responses, with a prospective phase to investigate individual variables through the use of a questionnaire. The antibody response after the COVID-19 vaccination was inversely related to old age, increased BMI and the number of smoking years, while a positive correlation was found with moderate alcohol consumption and especially with circulating levels of vitamin D, as clearly shown by the multivariate regression analysis. Our study showed that a number of variables are involved in the COVID-19 vaccine antibody response. These findings are very important and can be considered in the light of a future and personalized vaccinology.

## 1. Introduction

The COVID-19 pandemic has exerted a huge and complex impact on many different aspects of human health and society, not only in terms of morbidity and mortality, with more than 6 million deaths worldwide, but also with regard to economic and environmental aspects [1,2]. Vaccinations are certainly one of the most effective preventive measures against this infection [3]. Development of COVID-19 vaccines proceeded at a remarkable speed as, in general, between 4 to 15 years are required to have a safe and effective vaccine for human use. This goal was possible thanks to some crucial points, starting from the obtaining of the complete viral genome sequence of SARS-CoV-2 less than a month after the first documented cases in Wuhan [4]. Following this essential information, several laboratories worldwide have started to produce an effective SARS-CoV-2 vaccine.

mRNA vaccines have been the most important typology of performed and tested vaccines against COVID-19 [5]. Specifically, two different mRNA vaccines, Pfizer/BioNtech’s BNT162b2 and Moderna/NIAID’s mRNA-1273, received Emergency Use Authorization (EUA) status from the U.S. Food and Drug Administration (FDA) and other countries [6,7]. Specifically, Pfizer/BioNtech’s BNT162b2 is an mRNA–lipid nanoparticle-formulated vaccine encoding a membrane-bound, stabilized form of the full-length SARS-CoV-2 S protein [8], while Moderna/NIAID’s mRNA-1273 encodes a prefusion stabilized form of the SARS-CoV-2 S protein and is delivered by lipid-encapsulated nanoparticles [9]. Many studies have shown very high efficacy of these two different vaccines, with values of 95% for both of them [10,11,12].

However, many different factors can play a role in the individual response to vaccines. Age, gender, comorbidities and lifestyles, such as smoking habits, alcohol abuse and physical activity, are well-known factors that have an impact on immunity to natural infections and vaccinations [13]. The immune changes linked to age are on the basis of the higher severity of some viral and bacterial infections (e.g., influenza, herpes zoster, pneumococcal disease) often occurring among the elderly compared to younger people and also of the development of more common long-term sequelae [14]. Furthermore, the hormonal differences between sexes could explain the differences between women and men not only in the onset of autoimmune diseases but also in response to natural infections and vaccinations. However, in old age, the immunosenescence would be more pronounced in women than men due to menopause [15].

Finally, vitamin D circulating levels have been associated with good immune responses to natural infections and vaccinations [16,17]. Vitamin D is a steroid hormone produced in human skin from 7-dehydrocholesterol following exposure to sunlight ultraviolet B rays (UVB; 280–315 nm range) [18]. Skin production by sunlight exposure is the most important source of vitamin D, while principal nutrition sources are dairy products or fish liver oil [19]. The amount of melanin, reducing the penetration of UVB, decreases vitamin D skin production [18], and this has been shown to be linked to wide population differences in vitamin D synthesis after exposure to UVB [20]. In Europe, about 40% of the population is vitamin D deficient, with values lower than the minimum threshold of 20 ng/mL [21,22]. Similarly, approximately 24% of the U.S. population and 37% of Canadians have poor levels of vitamin D, especially in their non-white communities [22]. Various socioeconomic features can affect the levels of vitamin D, such as work characterized by sun exposure time, clothing habits influencing the surface area exposed to sunlight, and a diet including or lacking food rich in vitamin D [23].

The purpose of this study was to evaluate the potential role played by some demographic, anthropometric, behavioral, clinical and laboratory parameters in the production of antibody responses of a group of healthcare workers (HCWs) vaccinated with the complete primary cycle of anti-COVID-19 Pfizer/BioNtech’s BNT162b2 vaccine, in the light of a more and more personalized and future vaccinology.

## 2. Materials and Methods

We conducted an observational study to investigate the role of some variables on the antibody response to the COVID-19 vaccine. The study was conducted in accordance with the Declaration of Helsinki, and the protocol was approved by the local ethics committee (the University of Messina, protocol number 1860 of 05/10/2022).

The study involves a combination of a retrospective phase, exploiting previous data, with a prospective phase that aims to investigate demographic, clinical, behavioral and nutritional variables on already existing samples.

Specifically, we selected 200 people from a big sample of healthcare workers subjected to the primary cycle of the anti-COVID-19 Pfizer/BioNtech’s BNT162b2 vaccine, carried out between January and May 2021 in the University Hospital “G. Martino” of Messina, Italy. These selected people then underwent an evaluation of their antibody responses as part of routine health surveillance three weeks after the third dose of the vaccine. They possessed variable antibody responses and voluntarily adhered to the study. All the participants were asked to sign an informed consent for the study participation and the use of their serum/plasma samples stored at −80 °C in the virology laboratory of our hospital, followed by the self-administration of a questionnaire for the collection of information useful for evaluating any factors involved in the antibody response, such as their age, gender, body mass index (BMI), vitamin D intake, any comorbidities as well as persistent lifestyle aspects such as their smoking habits, alcohol consumption and physical activity. The information relating to lifestyles was collected using the indicators provided by the Italian PASSI surveillance system, recognized as a system of national importance [24].

The antibody response was evaluated, after centrifugation at 4000 rpm for 10 min, by CLIA test (ChemiLuminescence ImmunoAssay) (LIAISON SARS-CoV-2 S1/S2 IgG—DIASORIN SpA, Saluggia, Italy) consisting of a quantitative assay for the determination of IgG antibodies against SARS-CoV-2 S1/S2 antigens. Specifically, values <12 AU/mL were considered negative.

After excluding people who have contracted the COVID-19 infection and those who made use of vitamins and micronutrients, we proceeded to evaluate, on the same samples on which the antibody response had been determined, the quantitative determination of 25(OH)D3 plasma levels by high-performance liquid chromatography (HPLC) with a Bio-Rad 25(OH)D3/D2 kit (Bio-Rad, Milan, Italy) according to the manufacturer’s instructions. Separation of 25-OH-vitamin D3 and internal standard took place on a reversed-phase cartridge, followed by subsequent UV detection (λ = 265 nm) and quantitative evaluation [25].

The antibody titer was then correlated with biological parameters such as sex and age, comorbidities, behavioral factors and levels of vitamin D.

### Statistical Analyses

Statistical analyses were performed using Prism 4.0 software (GraphPad, San Diego, CA, USA). All the data obtained on the samples under study were subjected to a preliminary descriptive analysis aimed at summarizing the collected information. Pearson’s correlation test was used to determine any correlations between the studied variables. Stratified data were statistically analyzed using one-way ANOVA and *t*-tests. Finally, a multivariate analysis was carried out to evaluate the predictive capacity of the detected variables (demographic data, lifestyle, nutritional factors and clinical data) on the “antibody response” outcome. Analysis was performed by using the “a priori” model (i.e., considering as covariates all variables, regardless of *p* values to Pearson test) of multiple regression. Significance was assessed at the *p* < 0.05 level.

## 3. Results

The total number of the sample, the mean age, the gender percentages and all the information collected on the study participants related to antibody responses, body mass index (BMI), vitamin D levels, comorbidities and persistent smoking habits, alcohol consumption and physical activity are summarized in Table 1.

Of the initial sample number, 152 people were enrolled in the study. The mean antibody response of the primary vaccination cycle was 214.51 AU/mL (CI 95%: 187.4–245.2). Men had a slightly higher mean antibody response than women. However, no statistically significant difference was found in the antibody response between sexes, given a little difference of only 20 AU/mL in the antibody response.

**Figure 1:**
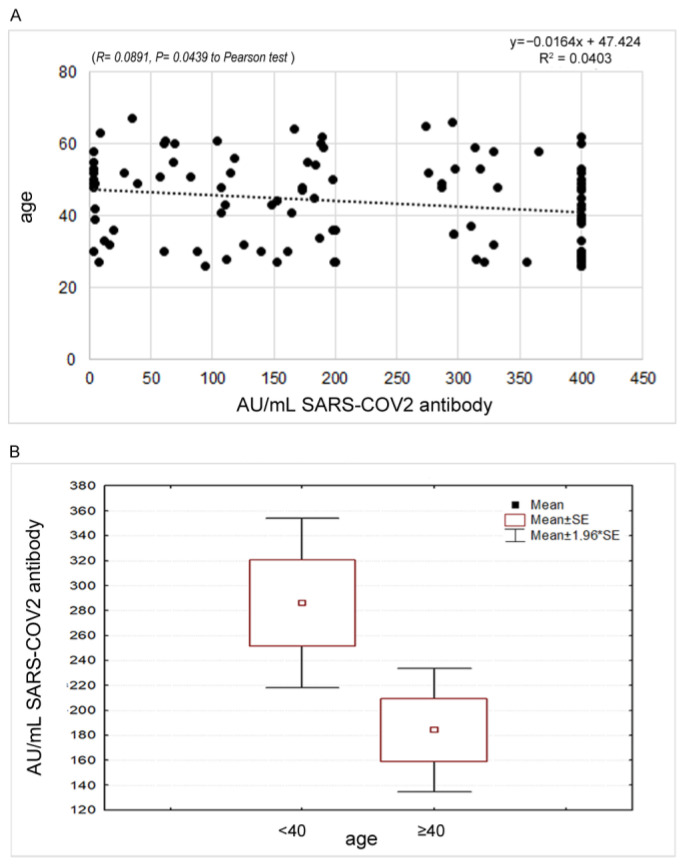
(**A**) Statistical correlation between the antibody response and the age of the sample. (**B**) Different expression of the antibody response among the group of people aged < 40 years compared to the group of people aged 40 years and older.

Conversely, a significant reverse correlation (*p* = 0.0439) was found between the antibody response and the age of the sample (Figure 1), with a decrease in the oldest age. A significant difference was observed stratifying the samples in <40 and ≥40 years old (286.2 ± 138.6 vs. 184.2 ± 138.4; t 2.37 *p* = 0.022).

**Figure 2:**
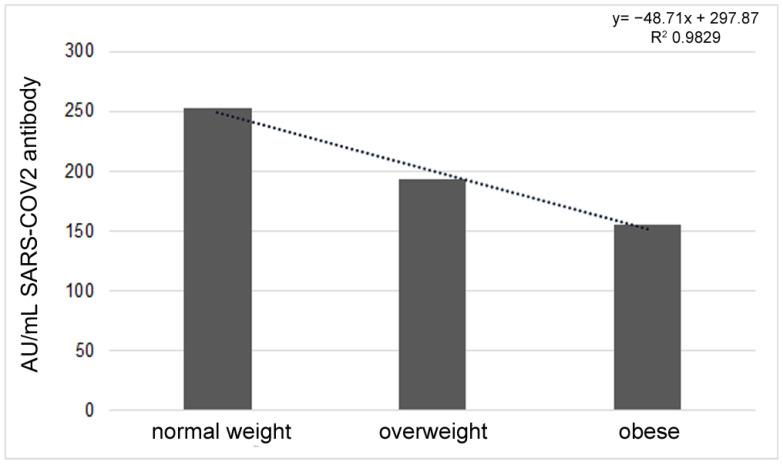
Different antibody titers according to BMI value, considering the three groups of normal weight, overweight and obese.

According to the BMI, whose mean value was 25.76 (CI 95%: 24.19–27.34), no significant correlation was found with the antibody response. Dividing the sample into three groups on the basis of the following BMI values: 18.5–24.99 = normal weight, 25–29.99 = overweight, ≥30 = obese [26], an interesting decrease in the antibody response was highlighted when BMI increased. However, the observed data of 252.87 ± 140.11AU/mL, 193.02 ± 139.44 AU/mL and 155.45 ± 162.78 AU/mL in the three groups, respectively (Figure 2), were not significant to the Anova test.

**Figure 3:**
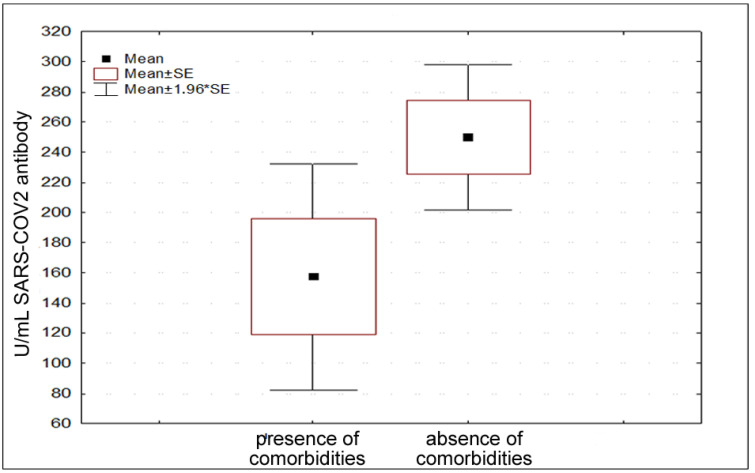
Different antibody titers considering presence and absence of comorbidities.

According to comorbidities, 33.33% of the participants declared their presence. Specifically, autoimmune/inflammatory diseases and hypertension were the most frequently declared ones. In this regard, a significant difference was found in antibody response in people with and without comorbidities (Figure 3) (t = 2.089, *p* = 0.0425).

**Figure 4:**
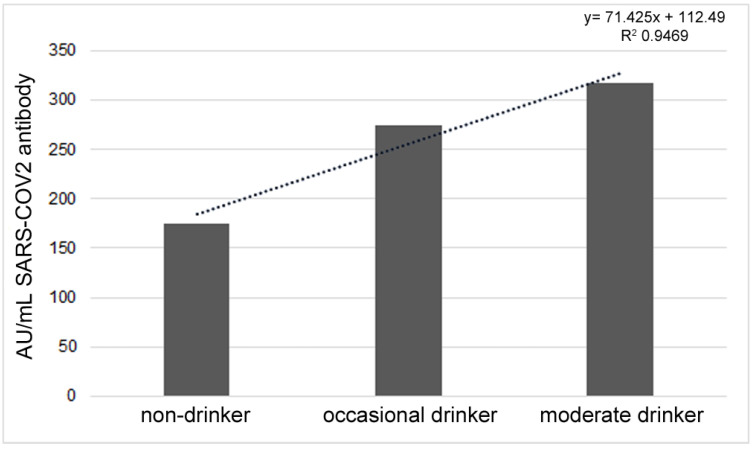
Different antibody responses in non-drinkers, occasional drinkers and moderate drinkers.

Regarding alcohol intake, 45.65% of the participants were drinkers, of which 23.91 were occasional drinkers and 21.74 were moderate drinkers; in both cases, the consumption of alcohol was declared to occur mainly during meals. None of the participants declared binge drinking or heavy drinking. The antibody response significantly increased with the frequency of alcohol consumption (R = 0.0856,*p* = 0.0484). By dividing the sample into three groups, non-drinkers (54.35%), occasional drinkers (23.91%) and moderate drinkers (21.74%), there were significant differences between groups (F = 4.069, p = 0.0248) (Figure 4).

According to physical activity, 73.33% of the subjects carried out moderate (42.22%) or light (31.11%) physical activity during work; moreover, 31.11% and 46.67% practiced intense and moderate physical activity, respectively, in their free time. However, no correlation was found between the antibody response and physical activity.

**Figure 5:**
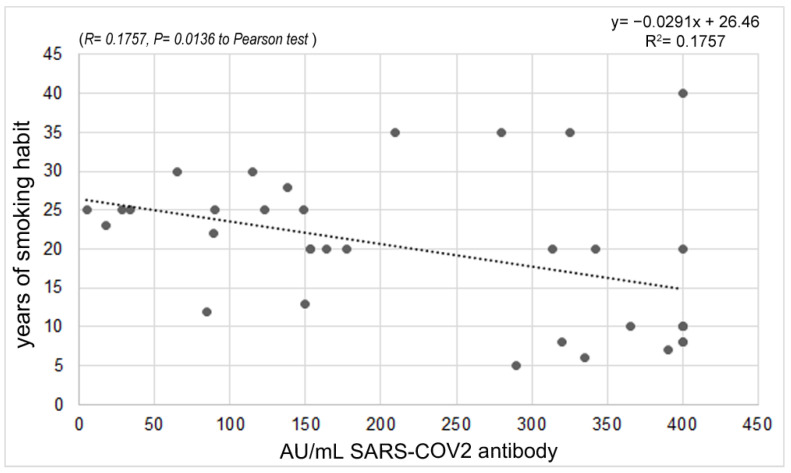
Correlation between antibody response and years of smoking.

Regarding cigarette smoking habits, 22.22% of the enrolled people were smokers, and 13.33% were former smokers. No statistically significant difference was found in the antibody response between smokers and non-smokers. However, considering the years of smoking habit, a significant reverse correlation (*p* = 0.0136) was found, likely due to the confounding factor of age (Figure 5).

**Figure 6:**
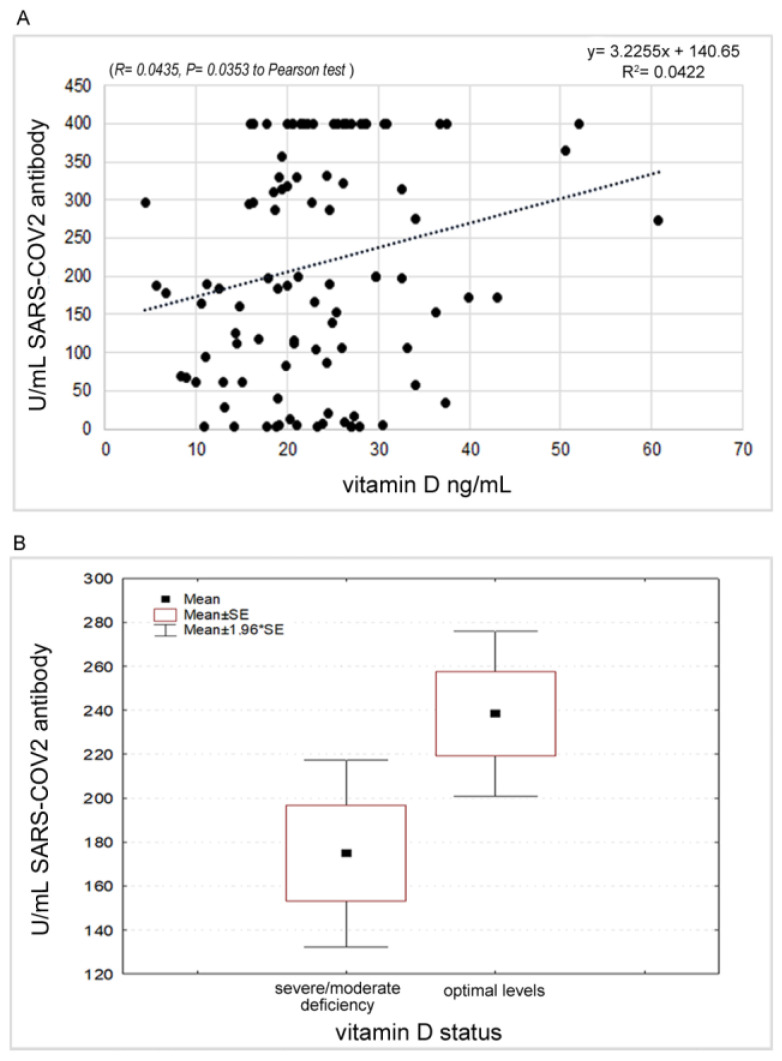
(**A**): Correlation between antibody response and Vitamin D values. (**B**): Different expression of the antibody response with respect to the Vitamin D status.

A significant positive correlation (*p* = 0.0353) was found between antibody response and vitamin D levels. The mean value of circulating vitamin D levels was 23.10 ng/mL (CI 95%: 21.24–24.97). Stratifying the sample according to the thresholds indicated as severe deficiency (<10), moderate deficiency (10–20) and optimal levels (21–75) [27], an increase in antibody responses equal to 143.80, 181.00 and 238.41, respectively, was found. The comparison between physiological and pathological levels (i.e., severe and moderate deficiency) highlighted a significant difference in the *t*-test (t = 2.109, *p* = 0.0375) (Figure 6A,B).

Finally, the multivariate analyses showed that, among the considered variables, only the plasma levels of Vitamin D were significantly related to the antibody response (*p* = 0.026), while no effects were shown by the demographic, anthropometric, clinical and behavioral variables (Table 2). As reported in Table 2, overall, nearly 37% of the observed variability in antibody response was due to vitamin D levels, showing the enhancer role of vitamin D in immune responses. Surprisingly, in our sample, the multivariate analysis did not show significant effects for the variables age and presence/absence of comorbidities, although the results of the bivariate analysis showed significant differences. On the other hand, the absence of variability in the antibody response attributable to smoking is plausible since, undoubtedly, the significant correlation in the bivariate analysis is due to collinearity with age since the elderly people have a consolidated and lasting exposure to smoke.

## 4. Discussion

Personalized vaccinology is the application of the idea of personalized medicine to vaccines. To date, after evaluating the absence of side effects, the target to reach is the immunization of the entire population using the same vaccine formulations with the same vaccination schedule for all individuals. This paradigm assumes that the same vaccine will elicit the same kind of antibody response in everyone, with similar levels of antibodies. A personalized vaccinology approach could upset the practice of vaccinology with great benefits for human health. In this research field, vaccinomics will allow us to understand the molecular immune predispositions of antibody responses to vaccines, with the possible development of early biomarkers of vaccine response, identify who should get vaccinated and how and increase safety and public confidence in vaccines [28]. A personalized vaccinology approach would suggest the development of specific vaccines based on several factors. In some cases, it can mean merely adjusting the dose based on weight, gender, or age. In some others, a deep study of a genetic predisposition to vaccine response is needed. As a result, a new era of personalized and predictive vaccinology able to design and develop new vaccines has to be reached in order to acquire the ability to give a vaccine based on the likelihood and need of response with the number of doses likely to be needed to induce a protective response to a vaccine [29].

This study was conducted to obtain information on the variability of antibody levels after the primary cycle of COVID-19 vaccination with respect to various demographic, anthropometric, behavioral, clinical and laboratory characteristics. The choice of the cohort fell on healthcare workers (HCWs), as they are one of the categories more at risk of contracting COVID-19 [30,31], considering the various cases of nosocomial transmission of SARS-CoV-2, which highlight the need for healthcare workers to strictly adhere to infection control measures in order to protect themselves and avoid transmission to hospitalized patients and nosocomial outbreaks [32]. Therefore, it is also important to identify variables that can influence the response of this category to vaccination.

One of the most important parameters that determine antibody response is certainly age, which represents an important response endogenous factor to natural infections and vaccinations. Specifically, as shown by our results and by previous scientific evidence, a natural decrease in the antibody response can be found with increasing age due to the physiological process of immunosenescence [13]. This process leads to a decrease in T-cell-derived antibody production and B-lymphocyte generation with advancing age, resulting in an often insufficient antibody response against infectious agents and following vaccination [33]. In the elderly, vaccine responses are often lower and frequently fail to induce long-lasting immunity, putting these individuals at risk of contracting infectious diseases [34]. In various serological studies carried out after vaccinations (i.e., against influenza, hepatitis A, hepatitis B, pneumococcal, tick-borne encephalitis, tetanus and SARS-CoV-2), it was observed that the post-vaccination antibody titer was inversely proportional to age [35,36,37]. In this view, it would be essential to consider age as one of the most important factors leading to a personalized vaccinology approach, for example, increasing the amount of the antigen or the number of doses to perform. Actually, this approach has already been partially followed in the development of a flu vaccine more specific for the elderly for its higher amount of antigen (high-dose flu quadrivalent vaccine containing a four-fold higher antigen dose compared to the other ones) [38].

Another important factor affecting the development of post-vaccine antibodies is gender. Although no correlation emerged in this study, our previous study [39] showed an increase in the antibody response in elderly women. In fact, it has been generally shown that the female immune system is more reactive during the youngest age than males, with more elevated circulating levels of antibodies following the positive influence exerted on the humoral immune responses by genetic and hormonal causes. As it is well-known, important genes involved in the innate and adaptive immune response to viral infections are located on the X chromosome [40,41]. In women, the X chromosome inactivation prevents the over-expression of X-linked genes, but some sequences, including those involved in the regulation of the immune function, can escape this process. As a consequence, these genes can double their expression, with predictable functional consequences represented by a better immune response to infections in women in their lifetime. Moreover, estrogens can enact these positive immunological effects [42,43]. Conversely, androgens would modulate immune responses negatively by affecting both the innate and the adaptive immune systems (immunosuppressive action) [44]. Both these aspects can explain the differences between women and men not only in the onset of autoimmune diseases but also in response to natural infections and vaccinations.

Even if no significant statistical difference was present between BMI and antibody response, our results found in this parameter a factor influencing antibody response after vaccination in terms of a decrease in antibody production with the increase of BMI. In line with this result, BMI has been identified as one of the factors influencing post-vaccine antibody production [45]. Moreover, it has been shown that obesity is a predisposition to SARS-CoV-2 infection compared to individuals with a normal BMI due to an abundant expression of the ACE2 receptor in adipose tissue [46]. Indeed, cytotoxic CD8 T-cell, CD4 T-helper, memory T-cell and antibody responses after vaccination are impaired in those who are obese in terms of BMI [13,46]. For all these considerations, special attention could be paid to people affected by obesity in terms of post-vaccination immune responses, trying to adapt to the current vaccination schedules and considering these groups of people are particularly at risk of not developing an effective immunity.

In addition, the presence of comorbidities influenced, in a negative way, the antibody response after vaccination. This finding is very important considering that some chronic pathologies, such as diabetes, cardiovascular diseases and autoimmune diseases, are particularly spread worldwide, especially in people of a certain age. This should be taken into particular consideration in terms of vaccination policies because we cannot consider people affected by comorbidities in the same way as healthy people. In this regard and in this kind of people, a personalized vaccinology approach should be stressed and improved.

A key role in the antibody response to vaccination has been identified in lifestyles, which influence the immune response to both natural infections and vaccinations. For example, concerning cigarette smoking, we found an inverse correlation between immune responses and years of smoking. Indeed, smoking has been shown to impair the overall immune response and the ability to form memory cells, necessary for long-lasting immunity, by causing a decrease in vaccine-induced IgG antibody levels [47,48]. In fact, many studies have shown that smoking can affect the immune response after hepatitis B vaccinations and flu vaccines, with a more rapid decrease in post-vaccination antibodies in smokers compared to non-smokers [49]. In this context, it will never be enough to emphasize the need to fight against smoking habits, especially in the youngest people, and the awareness of a lesser response to vaccinations and, in general, to natural infections in people who used to smoke can represent a further and important element to make the decision to stop smoking. Especially in heavy smokers, we can consider the need to change the currently used vaccination schedule, i.e., increasing the number of doses, in order to obtain a satisfying immune response.

Another very interesting finding of our study is the positive correlation found between the antibody response to the vaccination and the moderate consumption of alcohol. In fact, the consumption of alcoholic beverages can generally affect the immune response. Actually, there is strong evidence that chronic alcohol abuse is associated with increased immunosuppression and susceptibility to both bacterial and viral infections [50]. Conversely, moderate alcohol consumption exerts positive effects, which has been hypothesized to enhance vaccine response [51]. This increased response to vaccination is, according to Messaoudi et al. (2013) [52], due to an increased frequency of antigen-specific T cells and antibodies in moderate drinkers. In fact, alcoholic beverages such as beer or wine often contain resveratrol and/or group B vitamins. Polyphenols and particularly resveratrol (3,4,5-trans-trihydroxy-stilbene), a phenolic compound especially present in red wine, are natural compounds involved in maintaining health via the suppression of inflammatory reactions by acting on the immune cells [53,54]. They contribute to modulating innate and adaptive immunity stimulating the activation of macrophages, T cells and Natural Killer cells and cooperating in the inhibitory regulation of CD4+ CD25+ T cells [55]. In addition, group B vitamins represent essential micronutrients for a number of metabolic and regulatory processes; for example, they play an essential role in immune regulation [56].

Finally, a key role in the antibody response to vaccination has also been identified in vitamin D levels, a steroid hormone whose activity is mediated by the nuclear vitamin D receptors (VDRs) that, after activation and dimerization following the link with the vitamin, translocates to the nucleus to link the vitamin D receptor element (VDRE), a genetic element that regulates the expression of several host genes, some of them involved in immune responses like beta-defensin, which can directly split the outer viral membrane, and cathelicidins, which is involved in the activation of macrophages, dendritic cells and neutrophils [57,58]. Furthermore, vitamin D levels can affect the expression of toll-like receptors (TLRs), a class of proteins that play a key role in the innate immune system as they recognize pathogenic proteins, and of lysosomal enzymes also favoring the release of nitric oxide, both of which are implicated in the antimicrobial responses [59,60,61]. In addition, vitamin D affects T cell maturation shifting inflammatory versus anti-inflammatory responses decreasing the T helper type 17 (Th17) cell mass and increasing anti-inflammatory regulatory T cell (T-reg cell) populations. Therefore, vitamin D is able to reduce the levels of pro-inflammatory cytokines (IL-1, IL-6, IL-12, TNF alpha and IL-17) and increase the anti-inflammatory IL-10 [18,59,62]. All the above is perfectly in line with our results that found a strong positive correlation between antibody response to the COVID-19 vaccination and vitamin D levels, as also shown by the multivariate regression analysis that highlighted the leading role played by this vitamin in the protection against COVID-19 infection. In addition, Sadarangani et al. [16] reviewed some in vivo studies involving adult mice subcutaneously or intramuscularly vaccinated with inactivated vaccine co-administered with 1,25-(OH)2D3 and demonstrating the production of antigen-specific mucosal immunity (IgA and IgG antibodies), as well as enhanced systemic immune responses. The studies involved the inactivated polio vaccine (IPV), *Haemophilus influenzae* type b oligosaccharide conjugated to diphtheria toxoid vaccine and hepatitis B surface antigen (HBsAg). For all these reasons, it is reasonable to think that vitamin D supplements can play a crucial role in preventing respiratory infections, especially in people highly deficient in this metabolite, and in increasing immune responses after vaccinations [63]. Moreover, it appears of crucial importance to consider the impact of vitamin D and/or the VDR and vitamin D pathway gene polymorphisms on the immune response to vaccines in light of increasingly studied and pursued personalized vaccinology.

## 5. Conclusions

Our study showed that a number of variables are involved in the COVID-19 vaccine antibody response, some of them in a positive way and others in a negative way. Specifically, the antibody response after the primary cycle of mRNA COVID-19 vaccination was inversely related to old age, increased BMI and the number of smoking years, while a positive correlation was found with moderate alcohol consumption and especially with circulating levels of vitamin D. These findings are very important and can be considered in the light of a future and personalized vaccinology that can allow us to predict the efficacy of vaccination in a very accurate way in different groups of people depending on their demographic, anthropometric, clinical and laboratory parameters. These results could allow reaching higher vaccination coverage rates through increasing public confidence in vaccines following the improvement of their immunogenicity and the reduction of adverse event rates, with a reduction or elimination in the morbidity and mortality related to vaccine-preventable diseases.

## Figures and Tables

**Table 1:** Demographic, anthropometric, clinical and behavioral characteristics of the subjects included in the study.

	Total Sample	Women	Men
**Enrolled people**	152	58.82%	41.18%
**Mean age (± SD)** **(min–max)**	43.91 (±12.03) (26–67)	44.55 (±11.64) (26–67)	42.81 (±12.61) (26–64)
**Mean SARS-CoV2 antibody response (AU/mL)** **(min–max)**	214.51 (min 3.8; max ≥ 400)	209.23 (min 3.8; max ≥ 400)	229.02 (min 3.8; max ≥ 400)
**Mean BMI (±SD)** **(min–max)**	25.76 (±5.3) (18.82–44.92)	25.71 (±6.60) (18.82–44.92)	26.02 (±2.71) (22.86–32.91)
**Comorbidities (%)**	33.33%	34.61%	31.58%
**Physical activity at work**			
Moderate	42.22%	44.00%	36.84%
Mild/slight	31.11%	32.00%	31.58%
None	26.67%	24.00%	31.58%
**Intensive physical activity**	31.11%	11.5%	61%
**Light physical activity**	46.67%	42.31%	55.5%
**Smoking habits**			
smokers	22.22%	23.08%	22.22%
former smokers	13.33%	7.69%	21.05%
**Frequency of alcohol consumption**			
non-drinkers	54.35%	69.23%	36.84%
occasional drinkers	23.91%	19.23%	31.58%
moderate drinkers	21.74%	7.69%	31.58%
**Vitamin D (ng/mL)** **(min–max)**	23.10 (4.4–60.7)	23.62 (4.4–60.7)	21.47 (5.7–36.7)

**Table 2:** Multivariate regression analyses performed by using all the examined parameters (R2 = 0.310, *p* < 0.05) as independent variables associated with the immune response to the anti-COVID-19 vaccine.

	β Value	Standard Error of β Value	*p* Level
Gender	0.044	42.970	0.771
Age *	−0.234	1.748	0.123
BMI	0.089	4.098	0.560
Comorbidities	−0.237	44.135	0.116
Smoke	−0.005	0.174	0.971
Alcohol intake	0.282	18.541	0.074
Vitamin D *	0.366	2.085	0.026

* continuous variables.

## Data Availability

All data in this study have been included in the manuscript.
